# In Vivo Measurements of Transcranial Electrical Stimulation in Lesioned Human Brain: A Case Report

**DOI:** 10.3390/brainsci12111455

**Published:** 2022-10-27

**Authors:** Hongjie Jiang, Minmin Wang, Dan Wu, Jianmin Zhang, Shaomin Zhang

**Affiliations:** 1 Department of Neurosurgery, The Second Affiliated Hospital, Zhejiang University School of Medicine, Hangzhou 310030, China; 2 Clinical Research Center for Neurological Diseases of Zhejiang Province, Hangzhou 310030, China; 3 Key Laboratory of Biomedical Engineering of Education Ministry, Department of Biomedical Engineering, School of Biomedical Engineering and Instrument Science, Zhejiang University, Hangzhou 310027, China; 4 Binjiang Institute of Zhejiang University, Hangzhou 310051, China; 5 Qiushi Academy for Advanced Studies, Zhejiang University, Hangzhou 310027, China

**Keywords:** transcranial electrical stimulation, head model, in vivo, brain lesion, stereo-electroencephalography

## Abstract

Transcranial electrical stimulation (tES) has been utilized widely in populations with brain lesions, such as stroke patients. The tES-generated electric field (EF) within the brain is considered as one of the most important factors for physiological effects. However, it is still unclear how brain lesions may influence EF distribution induced by tES. In this case study, we reported in vivo measurements of EF in one epilepsy participant with brain lesions during different tES montages. With the in vivo EF data measured by implanted stereo-electroencephalography (sEEG) electrodes, the simulation model was investigated and validated. Our results demonstrate that the prediction ability of the current simulation model may be degraded in the lesioned human brain.

## 1. Introduction

Transcranial electrical stimulation (tES), as a promising non-invasive brain stimulation technique, has been widely utilized in various neuropsychiatric research, such as stroke rehabilitation [1,2] and epilepsy [3,4]. However, the clinical efficacy of tES is still inconsistent and varies substantially across subjects in practice [5,6]. EF generated by tES within the target brain regions is one of the most significant factors for physiological effects [7,8]. In addition, because individual brain lesions have different morphologies and tissue dielectric properties compared to normal brain tissue, the influence on induced EF may be different from that of normal tissues [9]. The individual variability would be further exacerbated in populations with brain lesions [10]. However, owing to the limitation of clinical practical methods, EF distributions are not easy to record directly from human brains, especially in patients with brain lesions.

Thus, computational modeling of tES has been proposed to predict specific spatial EF distribution within the brain in a given individual [11]. It is a good visual and quantitative tool for investigating the substantial inter-individual variability in the lesioned brain. There have also been some reports about individualized stroke head models [9,12,13]. Johnstone et al. reported the effect of brain lesions on tDCS-induced EF under various lesion locations, distances, sizes, and conductivities in a head model. They found that the lesion characteristics have a substantial effect on tDCS-induced EFs, such as that lesions can alter the tDCS-induced EF magnitude in a target by 30%. Notwithstanding, it is still highly controversial as to whether the simulation models could reliably reflect the actual situation, especially for the lesioned brain [9,14]. The actual measurement of EF in vivo is very important for the validation and optimization of the simulation model.

Several studies have reported on the measurement of tES-generated voltage in patients with epilepsy [15,16,17,18,19] or Parkinson’s disease [20,21]. In a previous study, the validation of a simulation model was also performed via in vivo measurements in epilepsy patients [16], but their data mainly came from implanted ECoG electrodes; the results may be influenced by skull defects caused by electrode implantation [22]. However, so far, all of these in vivo measurements were conducted in the brain without lesions. In vivo clinical evidence is still lacking in brain lesions [23]. Studying the influence of lesions on tES via in vivo measurements would lay a solid foundation for future clinical optimizations. 

In this case report, by using minimally invasive stereo-electroencephalography (sEEG) electrodes, we aim to conduct in vivo measurements of tES-induced EF in one epilepsy participant with brain lesions. Furthermore, the influence of brain lesions and electrode montage on EF were investigated, and the simulation model was validated with measured values.

## 2. Case Presentation

**Figure 1:**
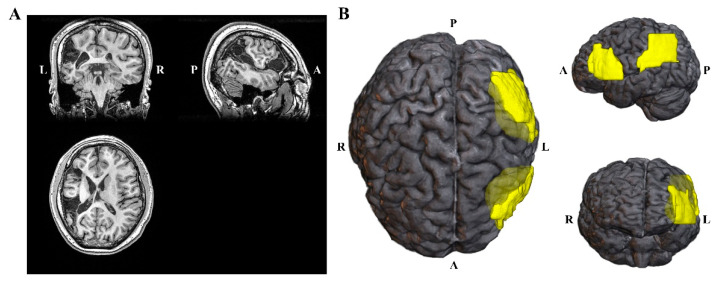
Three-dimensional (3D) MRI image and the locus of brain lesions. (**A**) T1-weighted MR brain images of participant. (**B**) Lesion map of participant, yellow areas represent brain lesions.

The participant was a 21-year-old, right-handed male who underwent sEEG recording to localize seizure foci for epilepsy surgery. As shown in Figure 1, he had two congenital malacia foci (yellow area in the right Figure 1B), one located in the left lateral middle and inferior frontal gyri, and the other located in the pars opercularis. His pre-implantation MRI data was acquired with a 3T MRI scanner (UIH Umr 790 system). The scanning parameters were TR = 8.2 ms, TE = 3.2 ms, flip angle =12°. Post-implantation CT data were acquired with a CT scanner (SIEMENS SOMATOM Perspective, 237 mA/ slice, 120 kV). The scanning resolution of the MRI and CT images was 0.5 × 0.5 × 1.0 mm^3^.

All clinical procedures were carried out in accordance with the Declaration of Helsinki. The Institutional Review Board at the Second Affiliated Hospital Zhejiang University School of Medicine approved the protocol (N2018-126). The participant has given his written consent before participating in the study.

## 3. Materials and Methods

### 3.1. Transcranial Alternating Current Stimulation

**Figure 2:**
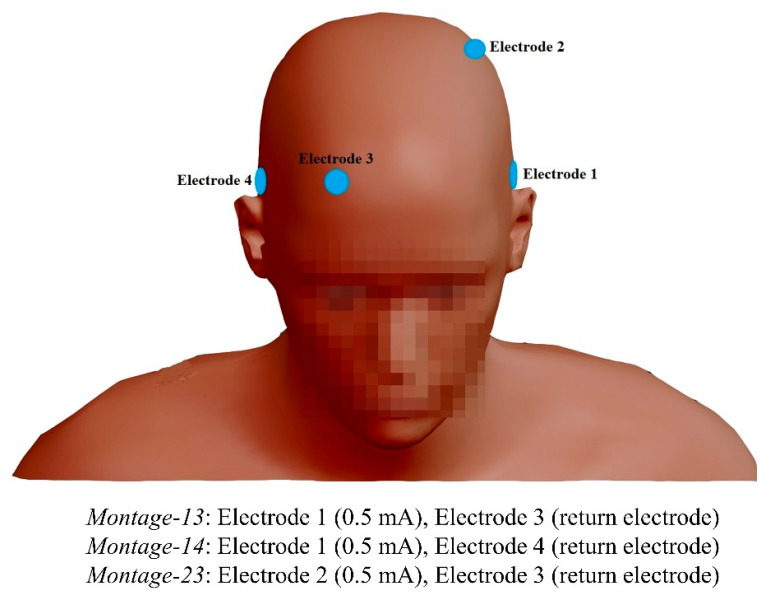
The positions of stimulation electrodes.

Four round Ag/AgCl electrodes (3.14 cm^2^, Pistim, Neuroelectrics, Barcelona, Spain) were attached to the scalp (T7, C3, Fp2, and T8, in order) and used in three different montages, as shown in Figure 2. At each stimulus trial, we applied transcranial alternating current stimulation (tACS) through two stimulation electrodes coated with a conductive gel (HD-GEL, Soterix Medical, New York, NY, USA), using a StarStim8 system (Neuroelectrics, Barcelona, Spain). 

Prior to each trial, we recorded a rest condition for 30 s and then applied 0.5 mA stimulation at a frequency of 100 Hz for 2 min with 10 s ramp up and down periods. Post-stimulation was recorded for another 30 s. The inter-stimulus interval lasted more than 30 s. For safety considerations, the impedance of the electrodes was monitored throughout the experiment and was always lower than 10 kΩ.

### 3.2. Intracranial Recording Setup

Based on clinical diagnosis, 112 sEEG recording electrodes (Sinovation, China; 0.8 mm diameter, 3.5 mm contact spacing, 2 mm contact length) were inserted to record the intracranial voltage changes around the suspected seizure foci. The locations and the numbers for the electrode insertions were determined for clinical reasons. The common reference for the recordings was the average value of two clinically adjacent recording electrodes (Electrode A5 and A6, located at the right inferior frontal gyrus). The ground electrode was positioned in the right mastoid region. The signal was band-pass filtered (0.16–300 Hz) using a clinical amplifier (EEG-1200C, Nihon Kohden, Tokyo, Japan) at a ~2 kHz sampling rate, and further filtered using a narrow band (95–105 Hz), zero phase, second-order Butterworth IIR filter to reduce noise interference. The tACS-induced average peak voltages were used as the measured value for subsequent analysis.

### 3.3. Model Simulations

**Figure 3:**
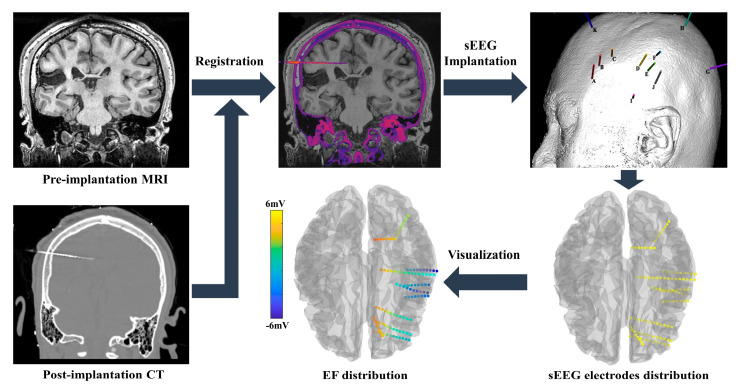
The analysis procedure of sEEG electrode positioning and Electric field modeling.

Our study adopted ROAST (Realistic Olumetric-Approach to Simulate Transcranial electric stimulation, ROAST 3.0, https://www.parralab.org/roast/ (accessed on 6 April 2021)) [24] for electric fields modeling. The sEEG electrodes were not modeled in EFs modeling (Figure 3). All tissues (including brain lesions) were assumed to have isotropic conductivities (WM: 0.126 S/m, GM: 0.276 S/m, CSF: 1.65 S/m, skull: 0.01 S/m, scalp: 0.465 S/m, brain lesions: 0.80 S/m [25], air cavities: 2.5 × 10^−14^ S/m, electrode: 5.9 × 10^7^ S/m, gel: 0.3 S/m). The MRI image was co-registered with a CT image, and each of the sEEG electrode locations were determined using Brainstorm software (brainstorm3, https://neuroimage.usc.edu/brainstorm/ (accessed on 2 November 2020)) [26]. Simulated voltage was obtained by re-referencing the sEEG recordings with the common reference.

### 3.4. Data Analysis

For data analysis, the projected electric field for both the predicted and measured values, was calculated by subtracting voltage values from adjacent electrodes and dividing by their distance. The electric field component, along with the direction of the measurement electrodes (projection of the electric field), were then estimated. Based on the results above, correlations between the predicted and measured values were studied (Pearson correlation coefficient). Using linear regression, the simulation model was evaluated for its accuracy in estimating voltage distributions and EF distributions.

## 4. Measured and Simulated Electric Field of tES

**Figure 4:**
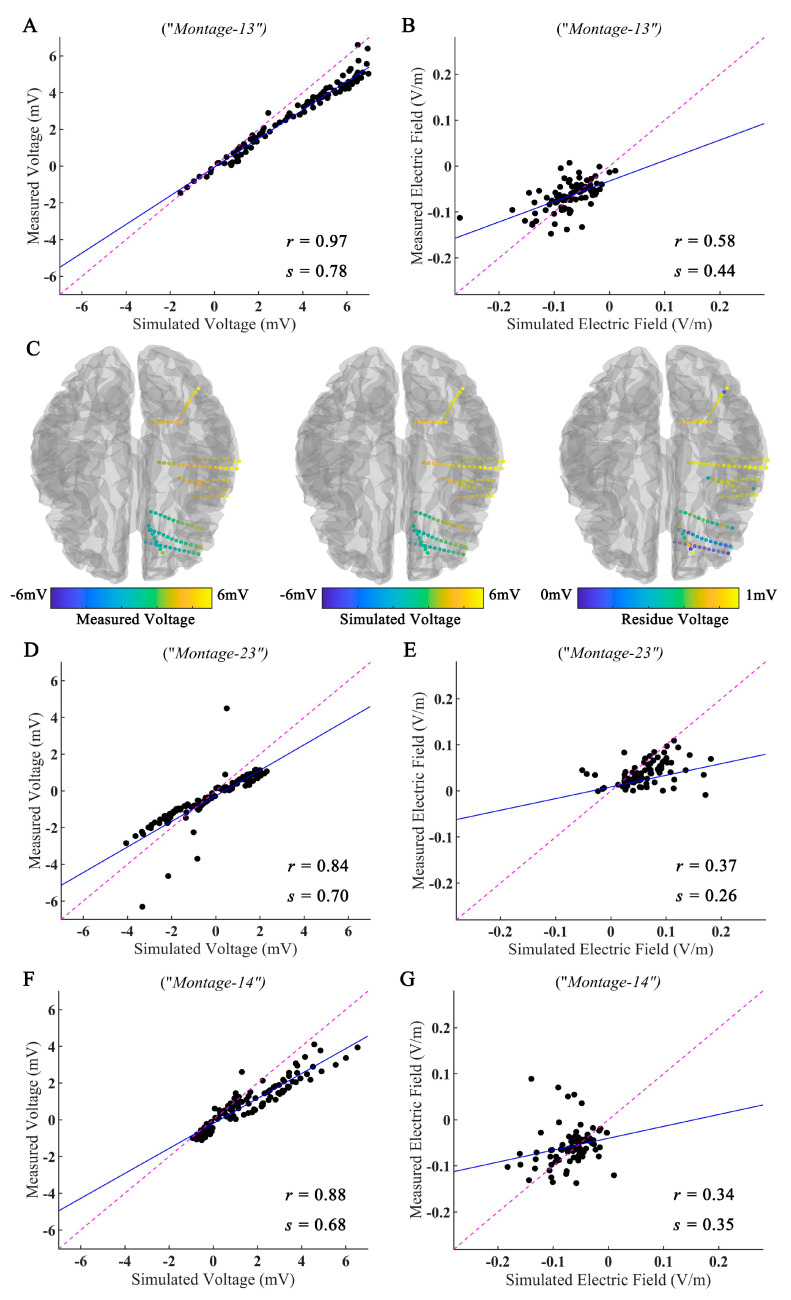
Measured and simulated electric fields of tES. (**A**,**B**) The measured voltages and projected electric fields with simulated values for *Montage-13*. (**C**) The spatial electric potential distributions from recorded and simulated voltages for *Montage-13*. (**D**–**G**) Measured and simulated results for *Montage-23* and *Montage-14*. Points falling on the magenta line represent perfect prediction (slope *s* = 1). The blue line represents the fitting line.

To investigate the influence of the electrode montage and brain lesion on the EF, we compared the simulation results with measured values. As shown in Figure 4A, the recorded voltages were highly correlated with the simulated voltage values (Pearson correlation coefficient *r* = 0.97, *p* < 0.001; slope of the best linear fit *s* = 0.78) under *Montage-13*. The spatial electric potential distributions from the recorded and simulated results for *Montage-13* are shown in Figure 4C. (The results for other montages are displayed in Figure S1.) As displayed in Figure 4B, we also found a moderate correlation between the measured and simulated values in the EF distributions (*r* = 0.58, *p* < 0.001; *s* = 0.44). In line with several previous studies [16,27], we found a lower correlation between measured and simulated EF, in contrast to voltage values.

Similar results were observed under *Montage-23* (Voltage: *r* = 0.84, *p* < 0.001, *s* = 0.70, Figure 4D; EF: *r* = 0.37, *p* < 0.001, *s* = 0.26, Figure 4E) and *Montage-14* (Voltage: *r* = 0.88, *p* < 0.001, *s* = 0.68, Figure 4F; EF: *r* = 34, *p* < 0.001, *s* = 0.35, Figure 4G).

## 5. Discussion

The case report, for the first time, provides direct evidence for tES-induced EF in the lesioned human brain. Several previous modeling studies have studied the effect of lesions on tES-induced EF [9,12,13,14,28,29,30]. These simulated results mainly explore the situation of stroke patients and have found that lesions caused a shunt of stimulation current in target areas. Although these simulation models have considered the effects of brain lesions, but have only provided computational results, it remains unclear as to how lesions may influence tES-induced EF within the brain. In this case report, our results show that the EF modeling can effectively predict EF distribution during tES in the lesioned brain under various electrical stimulation montages. The simulation model is also validated via in vivo measurement in previous studies of the non-lesioned brain [16,27]; they found a higher correlation between the simulated and predicted EF (Huang et al.: *r* = 0.89; Wang et al.: *r* = 0.73). These results indicated the influence of brain lesions on model performance. A variation in conductance may be one important factor. Lesions in the brain have a different conductance than healthy tissue [25], and the conductivity of different lesions is still unclear. In our simulation model, the conductivity of brain lesions (0.80 S/m) was assigned to be less than that of CSF (1.65 S/m). In our results, the simulated EF value seemed larger than the measured value, which may suggest that the conductivity value of the brain lesion is greater than 0.80 S/m. The EF is strongly influenced by large lesions that have a high assigned conductivity [12]. How to determine the conductivity of the lesion is essential for future research. 

It is noteworthy that the correlation of EF was lower than that of the voltage value. The observed dispersion in the electric field may come from several factors: First, the projected electric field was calculated in the direction of adjacent recording channels by subtracting the voltage values and dividing by their distance; thus, the amplitude of the electric field is relatively small, and the correlation of EF was decreased compared to the voltage. Second, the sEEG electrodes were not modeled in our simulation model, and the electric field near the electrode may be disturbed. Third, the influence of environmental noise, electrode contact, and the signal amplifier. Fourth, the influence of spontaneous brain activities, although this itself is relative small compared to the magnitude of the tES-induced voltage (Figure S2).

Limited to the nature of the clinical trial, our study only involves one lesioned human brain. However, lesion characteristics are variated in different individuals, such as distance and size. When lesions are larger, or closer to the target brain areas, the induced EF would have the greatest impact [30]. Thus, the characteristics of the lesion would exacerbate inter-individual variability in the current delivery [12]. More subjects are needed in future studies to investigate these variables. In addition, the simulation model would be improved when sEEG electrodes are modeled in a simulation model [28]. Our in vivo measurements provided precious data for studying the influence of lesions on tES-induced EF, which could shed light on future studies.

## Data Availability

Data involved in this study are available upon reasonable request.

## Supplementary Materials

The following supporting information can be downloaded at: https://www.mdpi.com/article/10.3390/brainsci12111455/s1. Figure S1: (A) The spatial electric potential distributions from recorded and simulated voltages for *Montage-23*. (B) The spatial electric potential distributions from recorded and simulated voltages for *Montage-14*. Figure S2: Example of signal analysis from one sEEG electrode. (A) Raw data from one sEEG electrode. (B) Band-pass filtered signal. (C) The power spectrum at each stimulation stage. (D) Time-frequency analysis based on wavelet transform.
