# Supplementary figures and images for: In Vivo Measurements of Transcranial Electrical Stimulation in Lesioned Human Brain: A Case Report
Source: Brain Sci. 2022 Oct 27;12(11):1455. doi: 10.3390/brainsci12111455 (PMC9688390; doi:10.3390/brainsci12111455)

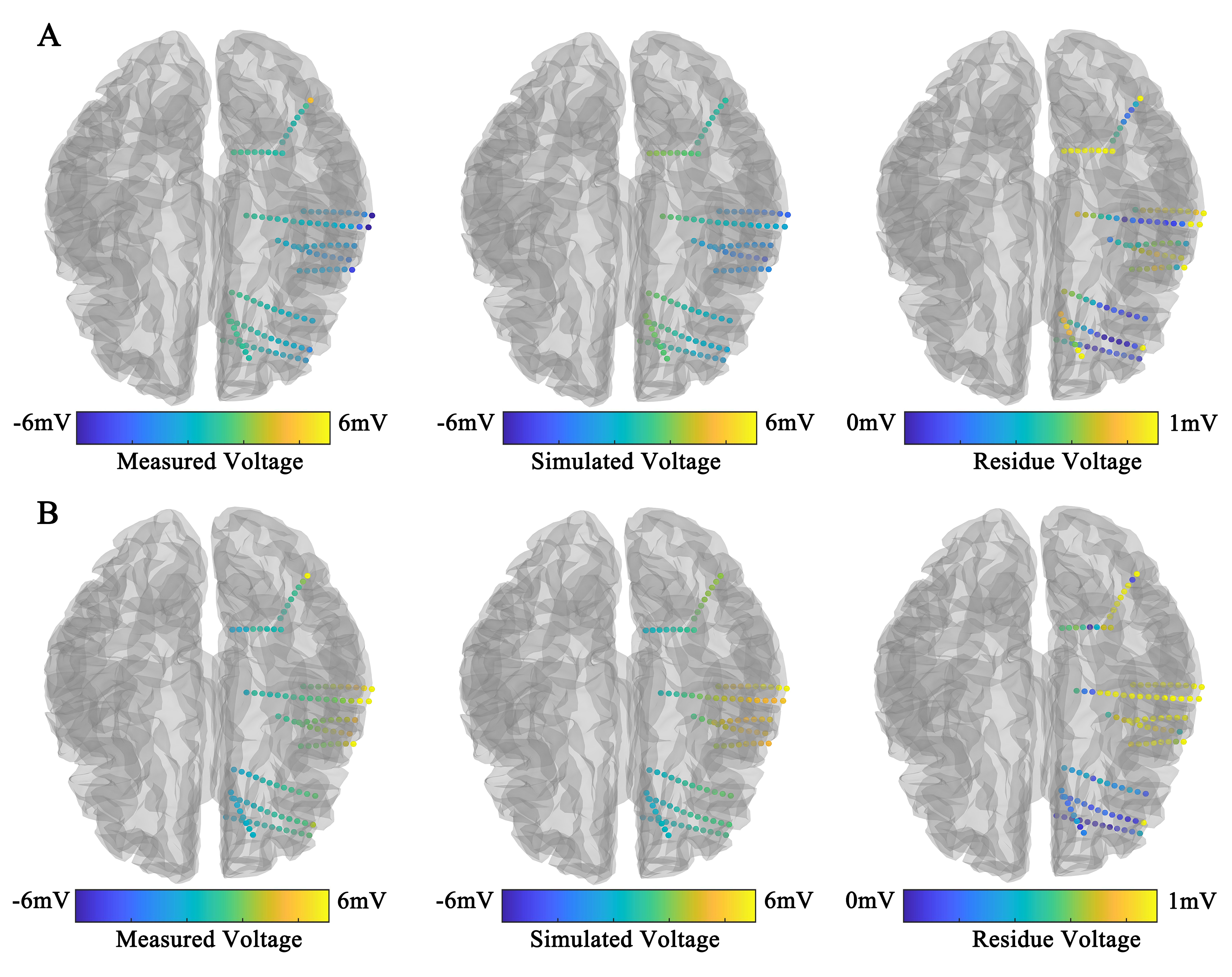

Supplement: Supplementary file 1 [file brainsci-12-01455-s001.zip › Figure S1.jpg]

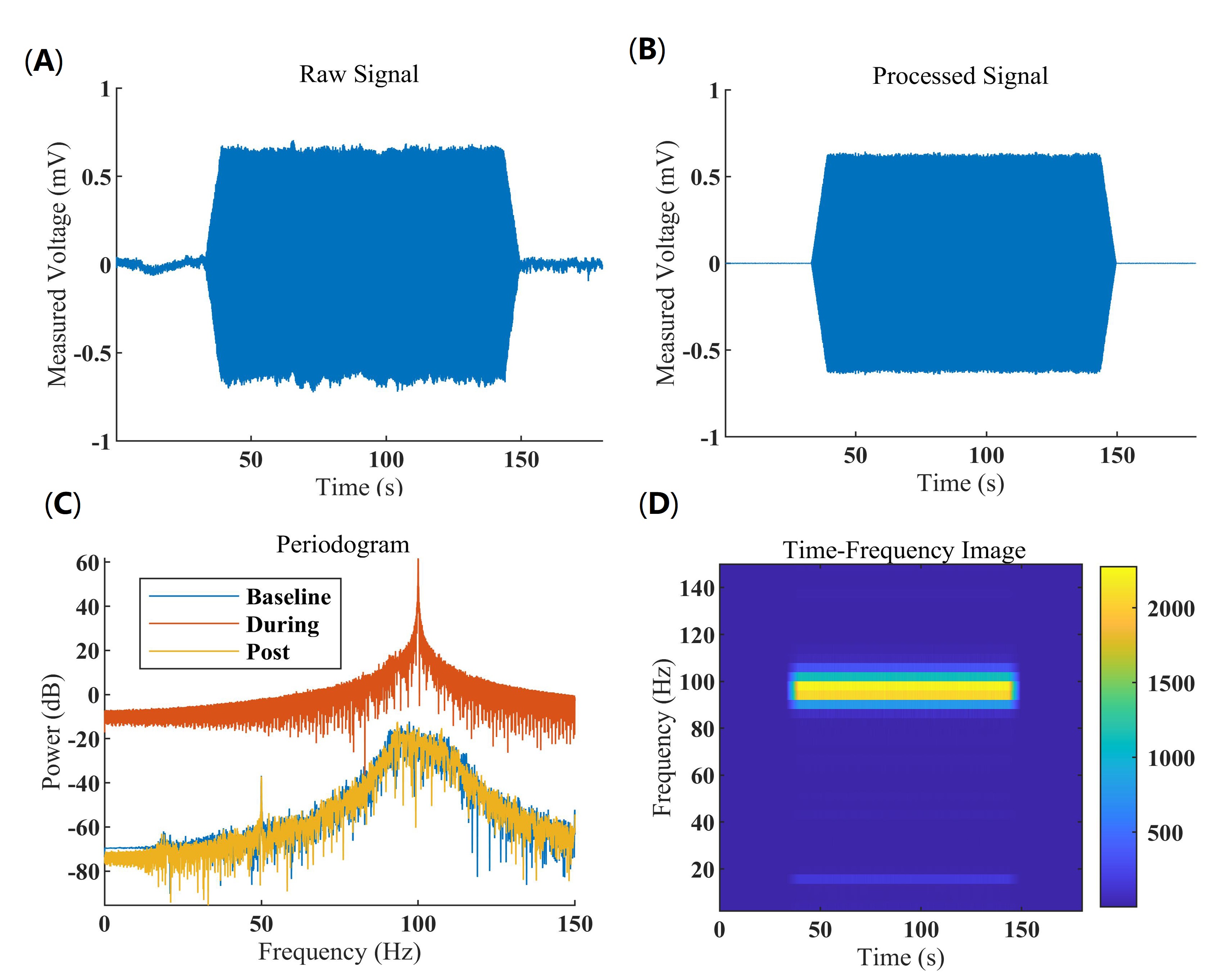

Supplement: Supplementary file 1 [file brainsci-12-01455-s001.zip › Figure S2.jpg]
